# Delayed Diagnosis of X-linked Lymphoproliferative Syndrome Type 2 in a 17-year-old Male With Severe Crohn’s Disease and Recurrent Skin Infections

**DOI:** 10.1097/PG9.0000000000000102

**Published:** 2021-07-12

**Authors:** Stephanie A. Zacharias, Priyanka Seshadri, Sharon Hwang, Laura Baker, Jonathan Powell, J. Fernando del Rosario, Zarela Molle-Rios

**Affiliations:** From the * Division of Gastroenterology and Nutrition, Nemours/Alfred I. duPont Hospital for Children, Wilmington, DE; † Sidney Kimmel College of Medicine at Thomas Jefferson University, Philadelphia, PA.

**Keywords:** *XIAP* deficiency, primary immunodeficiency disease, hematopoietic stem cell transplantation

## Abstract

X-linked lymphoproliferative syndrome type 2 (XLP2) is a rare genetic primary immunodeficiency disease caused by mutations in the *XIAP* gene that lead to deficiency of the X-linked inhibitor of apoptosis protein. XLP2 is characterized by dysregulated immune responses and can result in an inflammatory bowel disease (IBD)-like phenotype, a form of monogenic IBD. Patients with XLP2 often succumb to fulminant hemophagocytic lymphohistiocytosis or Epstein-Barr virus infections. Hematopoietic stem cell transplantation (HSCT) is currently the only definitive treatment for XLP2. We report an adolescent with a delayed diagnosis of XLP2 in the setting of severe Crohn’s disease diagnosed at age 9 years and recurrent skin infections. He is under evaluation for HSCT. Gastroenterologists must recognize monogenic IBD in patients of all ages with severe disease and signs of an underlying primary immunodeficiency disease. Patients with suspected monogenic IBD should undergo immunologic and genetic analysis at diagnosis to initiate potentially life-saving treatment.

## INTRODUCTION

X-linked lymphoproliferative syndrome type 2 (XLP2) is a rare genetic primary immunodeficiency disease (PIDD) caused by loss-of-function mutations in the *XIAP* (also known as *BIRC4*) gene that lead to deficiency of the X-linked inhibitor of apoptosis protein (1). The *XIAP* gene product functions as an apoptosis inhibitor and is also involved in a variety of signaling pathways and cellular responses, including nucleotide-binding oligomerization domain proteins 1 and 2 receptor signaling (1–3). Nucleotide-binding oligomerization domain protein 2 is the strongest susceptibility gene associated with Crohn’s disease (1–3), and the encoded receptor plays a pivotal role in innate immune host defense in the gut, including recognition of bacterial peptidoglycan products (1,2).

XLP2 is characterized by dysregulated immune responses and can result in a variable phenotype. Classic disorders include hemophagocytic lymphohistiocytosis (HLH) with or without Epstein-Barr virus (EBV) infection, splenomegaly, dysgammaglobulinemia, cytopenias, recurrent infections, and chronic colitis (1–3). Development of an inflammatory bowel disease (IBD)-like phenotype occurs in 25% to 30% of patients with *XIAP* deficiency (1) and is associated with poor outcomes (3). Aside from their IBD presentation, patients sometimes have no other signs or symptoms of XLP2; symptoms can be severe and are often resistant to standard treatment (1,2). Patients with XLP2 often succumb to fulminant HLH or EBV infections. Hematopoietic stem cell transplantation (HSCT) is currently the only definitive treatment for XLP2 (1–3).

Consent was obtained from our adult patient for publication of the case details.

## CASE REPORT

**FIGURE 1.**
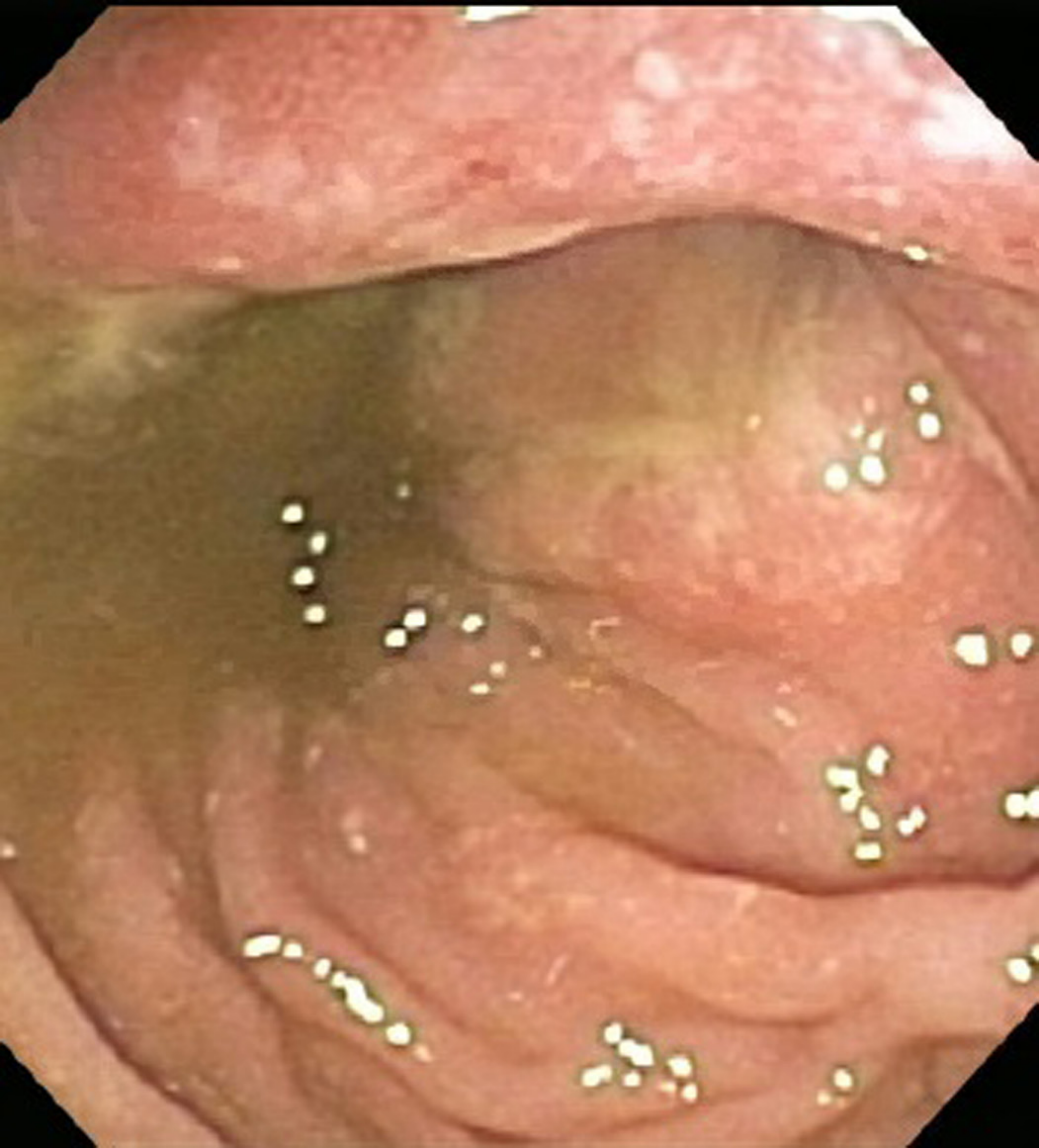
Initial colonoscopy revealed diffuse aphthae throughout the colon and in the terminal ileum as shown here in the rectum.

**FIGURE 2.**
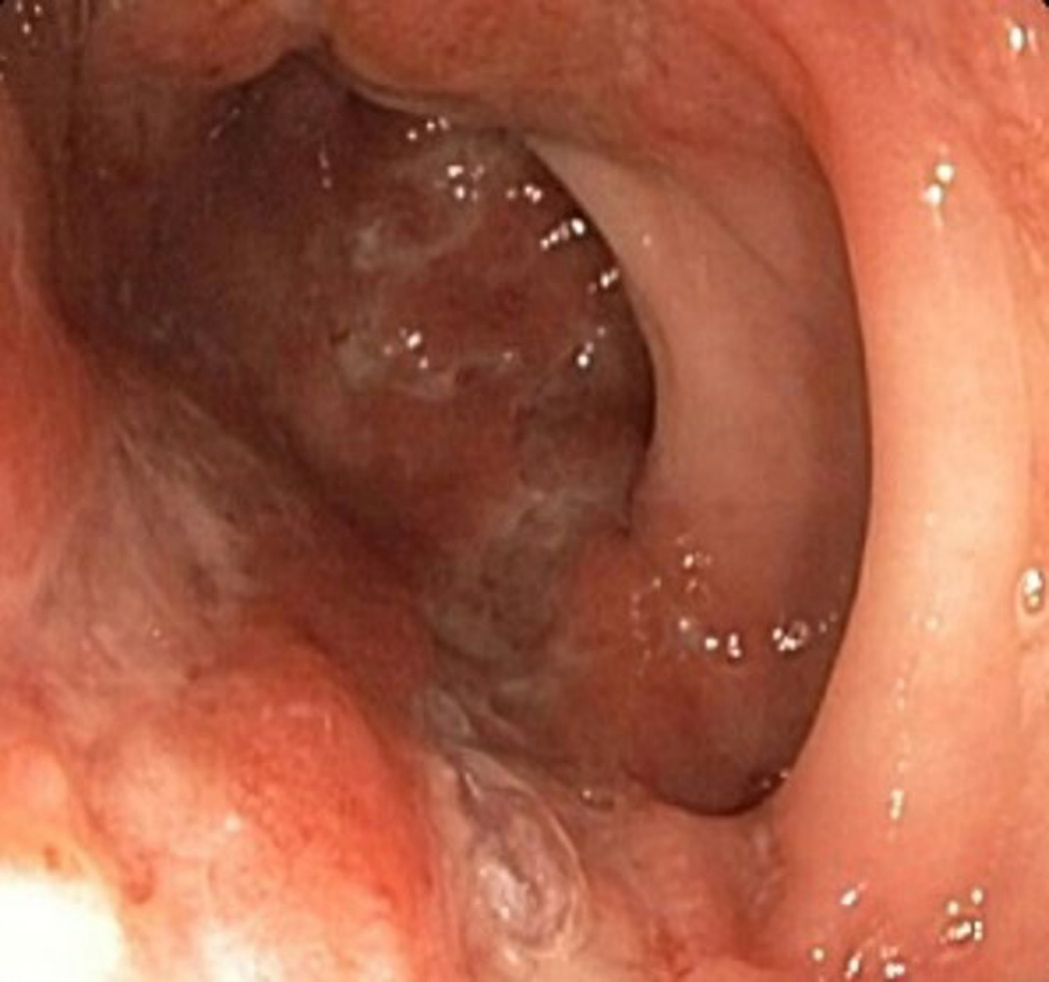
Repeat colonoscopy revealed multiple colonic areas of large stellate, moderately deep ulcers as shown here in the sigmoid colon.

This report describes a case of a 17-year-old male with a delayed diagnosis of XLP2 in the setting of severe Crohn’s disease and recurrent skin infections. He had a history of a pediatric intensive care unit admission as a toddler for atypical febrile seizures and pancytopenia, as well as a history of childhood sinusitis and recurrent skin lesions including psoriasis, folliculitis, and methicillin-sensitive *Staphylococcus aureus* abscesses. At the age of 9 years, he presented with periumbilical abdominal pain, rectal pain, and hematochezia; he was diagnosed with ileocolonic Crohn’s disease with upper gastrointestinal involvement and perianal skin tag. On esophagogastroduodenoscopy, he had multiple apthae in the stomach and an ulcer in the duodenal bulb. Colonoscopy revealed a perianal skin tag and diffuse apthae from rectum to terminal ileum in a skip distribution (Fig. 1). Pathology showed chronic active gastritis, duodenitis, and colitis with no granulomas, most consistent with Crohn’s disease. Initially, he was started on mesalamine and prednisone with a taper. Due to persistent hematochezia, methotrexate and rectal hydrocortisone were added to his regimen. Five months later, a repeat colonoscopy showed evidence of worsening colitis with enlarged perianal skin tags and several colonic areas of large stellate, moderately deep ulcers (Fig. 2). For the past 8 years, his colitis has been fairly controlled with an intensified infliximab regimen and oral methotrexate.

**FIGURE 3.**
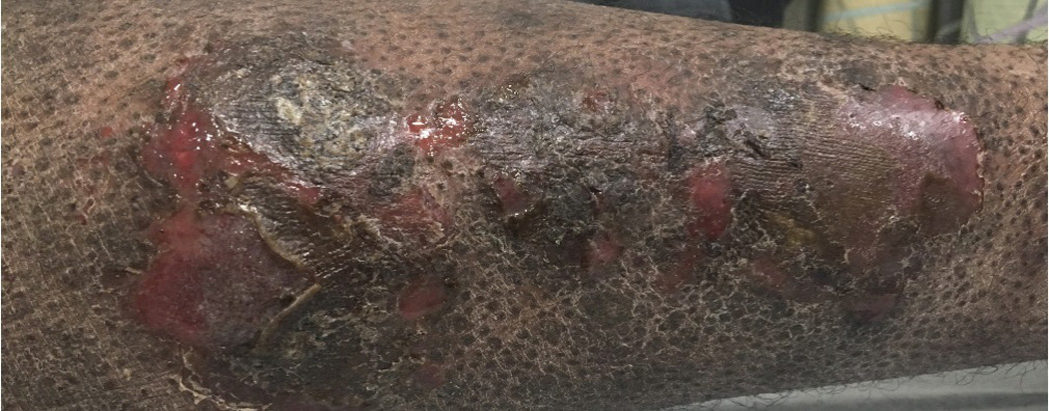
Patient developed severe cellulitis of the leg as shown here, resulting in admission for intravenous antibiotics and prompting extensive immunologic work up, which ultimately revealed the new diagnosis of X-linked lymphoproliferative syndrome 2.

During a recent hospital admission for severe cellulitis of the leg (Fig. 3), he underwent an extensive immunologic work up due to high suspicion for an underlying PIDD. The work up revealed dysgammaglobulinemia with elevated immunoglobulin G and immunoglobulin A levels, protective vaccine antigen-specific antibody levels, normal dihydrorhodamine test, normal T- and B-cell counts, normal T-lymphocyte mitogen stimulation test, and normal marrow flow cytometry. He had low natural killer cell counts with preserved natural killer function as shown by normal CD107a and perforin/granzyme B studies. XIAP flow cytometry demonstrated notably low XIAP expression. A next generation sequencing panel including genes related to monogenic IBD/PIDD revealed a pathogenic hemizygous mutation (c.894_898del; p.Lys299Leufs*9) of the *XIAP* gene, consistent with XLP2; this mutation was determined to be de novo after subsequent testing of immediate family. His EBV titers were consistent with past EBV infection; EBV DNA was detected by polymerase chain reaction with 3748 copies/mL in January 2020. He started sulfamethoxazole/trimethoprim for opportunistic infection prophylaxis and received rituximab infusions for EBV prophylaxis. After extensive discussions with the family, he commenced evaluation for HSCT.

## DISCUSSION

Pediatric-onset IBD accounts for about 25% of all IBD cases. Patients diagnosed with IBD before the age of 6 years are classified as very early-onset IBD (VEO-IBD); this comprises about 15% of all pediatric IBD cases. Patients with VEO-IBD exhibit an increased prevalence of monogenic causes of IBD, which are associated with defects in over 70 reported genes including the *XIAP* gene (3,4). Our patient was diagnosed with IBD at age 9 years and would be classified as early-onset pediatric IBD, defined as patients with IBD onset before 10 years of age (3).

In review of the literature, there are over 70 reported cases of patients with *XIAP* deficiency and IBD (1–10). Most of these cases involved much younger patients with VEO-IBD, with severe presentations and morbidity. Several of these patients succumbed to fulminant HLH (3).

Although our patient was not classified as VEO-IBD, there were early indications of a potentially dysregulated immune system given his history of a pediatric intensive care unit admission as a toddler with pancytopenia, his history of recurrent skin infections, and his severe Crohn’s disease diagnosed at age 9 years. This unusual past medical history should prompt a gastroenterologist to perform immunologic and genetic analysis for monogenic IBD. Our patient was 17 years old when he was eventually diagnosed with XLP2. In patients with XLP2 and severe colitis, HSCT should be considered early in the disease course to successfully treat intestinal inflammation and decrease the risk of developing HLH, which is associated with high mortality. By the age of 20 years, over 80% of patients with XLP2 typically develop HLH (3). At the time of this reporting, our patient did not develop fulminant HLH despite evidence of a prior EBV infection.

This case highlights the importance for gastroenterologists to have high suspicion for monogenic IBD in patients of all ages with a severe disease course and clinical signs of underlying PIDD. With more readily available genetic panels, testing for mutations responsible for monogenic IBD should be done at the time of diagnosis for suspected patients because identifying these patients early could result in potentially life-saving treatment, such as HSCT.
